# Continuous noninvasive monitoring of arterial pressure using the vascular unloading technique in comparison to the invasive gold standard in elderly comorbid patients: A prospective observational study

**DOI:** 10.1002/hsr2.204

**Published:** 2020-11-10

**Authors:** Phil Klose, Ulf Lorenzen, Rouven Berndt, Christoph Borzikowsky, Moritz Hill, Matthias Gruenewald, Gunnar Elke, Jochen Renner

**Affiliations:** ^1^ Department of Anaesthesiology and Intensive Care Medicine University Medical Center Schleswig‐Holstein, Campus Kiel Kiel Germany; ^2^ Department of Cardiovascular Surgery University Medical Center Schleswig‐Holstein, Campus Kiel Kiel Germany; ^3^ Institute of Medical Informatics and Statistics Christian‐Albrechts‐University Kiel, University Medical Center Schleswig‐Holstein, Campus Kiel Kiel Germany; ^4^ Department of Anesthesiology Helios Kliniken Schwerin Schwerin Germany

**Keywords:** arterial pressure, hemodynamic monitoring, orthopedic surgery, vascular surgery, volume clamp method

## Abstract

**Background and Aims:**

Elderly patients aged ≥65 years represent a growing population in the perioperative field, particularly orthopedic and vascular surgery. The higher degree of age‐related or comorbid‐dependent vascular alterations renders these patients at risk for hemodynamic complications and likely denote a possible limitation for modern, non‐invasive arterial pressure monitoring devices. The aim was to compare vascular unloading technique‐derived to invasive measurements of systolic (SAP), diastolic (DAP), and mean arterial pressure (MAP) in elderly perioperative patients.

**Methods:**

This prospective observational study included patients aged ≥65 years scheduled for orthopedic and patients ≥50 years with peripheral artery disease Fontaine stage ≥ II scheduled for vascular surgery, respectively. Invasive radial artery and non‐invasive finger‐cuff (Nexfin system) arterial pressures were recorded before and after induction of general anesthesia and during surgery. Correlation, Bland‐Altman, and concordance analyses were performed. Measurements of arterial pressure were also compared during intraoperative hypotension (MAP <70 mm Hg) and hypertension (MAP >105 mm Hg).

**Results:**

Sixty patients with orthopedic (N = 25, mean (SD) age 77 (5) years) and vascular surgery (N = 35, age 69 [10] years) were enrolled. Seven hundred data pairs of all patients were analysed and pooled bias and percentage error were: SAP: 14.43 mm Hg, 43.79%; DAP: −2.40 mm Hg, 53.78% and MAP: 1.73 mm Hg, 45.05%. Concordance rates were 84.01% for SAP, 77.87% for DAP, and 86.47% for MAP. Predefined criteria for interchangeability of absolute and trending values could neither be reached in the overall nor in the subgroup analyses orthopedic vs vascular surgery. During hypertension, percentage error was found to be lowest for all pressure values, still not reaching predefined criteria.

**Conclusion:**

Arterial pressure monitoring with the vascular unloading technique did not reach criteria of interchangeability for absolute and trending values. Nevertheless, the putatively beneficial use of noninvasive arterial pressure measurements should be further evaluated in the elderly perioperative patient.

## 1 BACKGROUND AND AIMS

The proportion of elderly patients aged ≥65 years is steadily increasing in the perioperative, particularly orthopedic, and vascular surgical setting.^1^, ^2^, ^3^ These patients generally exhibit a higher level of aging‐related cardiovascular alterations including atherosclerosis.^4^, ^5^ As this cohort may be particular susceptible to hyoptension during anesthesia, sufficient perioperative hemodynamic monitoring is a central task for patient safety.^6^, ^7^ Blood pressure measurement is one key factor and is most commonly performed using the the noninvasive oscillometric method with an inflatable cuff at the upper limb.^8^ However, the gold standard is the continuous invasive beat to beat blood pressure monitoring using an arterial line.^9^ An attractive alternative are completely noninvasive continuous monitoring devices using the “vascular‐unloading technique,” introduced by Penaz.^10^ This method records the pulse wave of the peripheral arterial blood volume by an optical plethysmograph mounted in an inflatable finger cuff^11^ and has the advantage of providing continuous arterial pressure measurements without the inherent risk of invasive monitoring.^12^

This noninvasive technique using different devices has already been investigated for its valid application for blood pressure monitoring in different clinical settings and patient categories, compared to either invasive or noninvasive blood pressure measurement.^13^, ^14^, ^15^, ^16^, ^17^ Poor performance of noninvasive finger blood pressure monitoring was mostly related to critically ill patients and clinical situations with reduced perfusion due to severe hypotension, disease‐related peripheral oedema, use of vasopressors or hypothermia.^18^, ^19^ Furthermore, an early study described age‐dependent differences in the clinical reliabilty of arterial pressure measurements due to a degenerative decline in peripheral reflection coefficient resulting from decreased distensibility of peripheral arteries.^20^ However, information regarding the application of noninvasive monitoring devices specifically in the group of elderly patients in the perioperative setting is limited. The higher degree of age‐related or comorbid‐dependent vascular alterations in the elderly likely denotes a possible limitation of the vascular‐unloading technique.^21^, ^22^

Therefore, the aim of the presented study was to test the interchangeability of blood pressure measurements using the vascular unloading technique (Nexfin finger‐cuff device) compared to the invasive gold standard of an arterial line in elderly patients scheduled for orthopedic and vascular surgery.

## 2 METHODS

### 2.1 Study design, patients, and ethical considerations

This was a prospective observational cohort study conducted from October 2015 to June 2017 at the Department of Anaesthesiology and Intensive Care Medicine, University Medical Center, Schleswig‐Holstein, Campus Kiel, Kiel, Germany. Inclusion criteria were defined as patients with an age ≥65 years scheduled for orthopedic surgery and ≥50 years with preexisting peripheral artery disease Fontaine stage ≥ II scheduled for (elective or urgent) vascular surgery, respectively. Further inclusion criteria were an American Society of Anesthesiologists (ASA) physical status classification II‐IV and written informed consent for study participation. Exclusion criteria were defined as need for vasopressor support prior to surgery, ASA physical status classification V, cognitive or linguistic barriers and emergency surgery. Types of orthopedic (eg, hip or knee replacement) and vascular surgery (eg, carotid and femoral endarterectomies or vascular bypass) were not further restricted in order to cover a broad spectrum of possible different hemodynamic changes associated with surgical‐ and anesthesia‐related hemodynamic changes and carotid baroreflex sensitivity. The study protocol was approved by the local ethics committee of the Christian‐Albrechts‐University Kiel, Germany (file number: A 135/14) and retrospectively registered with the trial registration number NCT03178097 at https://www.clinicaltrials.gov. Written informed consent was obtained from all patients prior to study participation. All study procedures were performed in accordance with the ethical standards of the institutional research committee and with the 1964 Helsinki declaration and its later amendments.

### 2.2 Intrumentation and study protocol

The standard anesthesia monitoring was established as follows: pulse oximetry, electrocardiography, and noninvasive blood pressure measurement taken by a cuff at the upper limb. The arterial line (Arrow R Intl., Reading, Pennsylvania; Transducer: DPT‐6000, CODAN pvb Critical Care GmbH, Forstinning, Germany) was placed under local anesthesia in Seldinger technique in the radial artery on the same side as the finger cuff, in accordance to previous study protocols.^15^, ^23^, ^24^ All patients were then connected to the noninvasive Nexfin monitoring system (BMEYE, Amsterdam, The Netherlands) being recently distributed as the Clearsight system (Edwards Lifesciences, Irvine, California).^25^ The technique of this finger cuff‐based device has been described in detail before.^11^ The correct size of the finger cuff belonging to the system was choosen and placed at the middle phalanx of the index finger ipsilateral to the reference arterial line and connected to the wrist unit and heart reference system. This system adjusts the blood pressure to hydrostatic differences between the sensor and the heart level. The Nexfin monitor was connected to the wrist unit and the measurement procedure started in accordance to the user manual. Obvious artifacts of the invasive and noninvasive‐derived arterial pressure measurements were excluded after visual inspection of the arterial pressure waveforms.

### 2.3 Data collection

**FIGURE 1:**
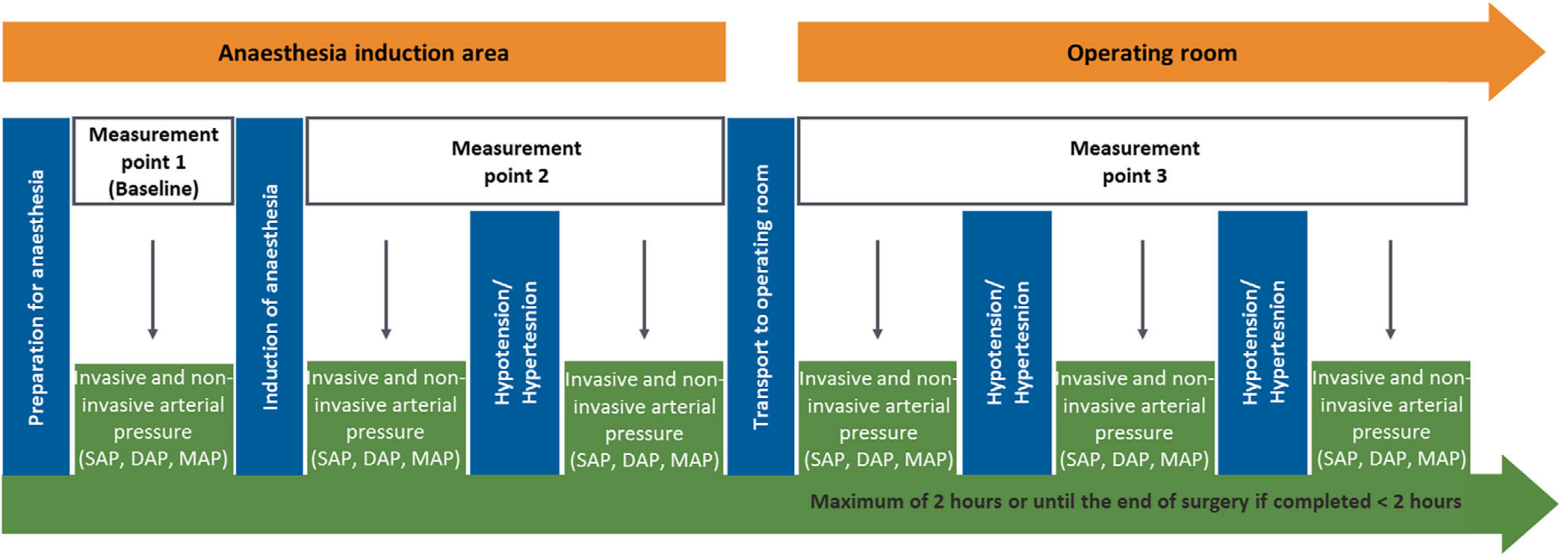
Study protocol with corresponding hemodynamic measurement time points. DAP, diastolic arterial pressure; MAP, mean arterial pressure; SAP, systolic arterial pressure

The study protocol is illustrated in Figure 1. After all monitoring devices were established, a first data sample of systolic (SAP), diastolic (DAP), and mean arterial pressure (MAP) was recorded generating baseline values for both devices in the awake, spontaneously breathing patient before induction of anesthesia (measurement time point 1). After induction of general anesthesia, measurement time point 2 was recorded. During surgery, measurements were taken every 15 minutes for a maximum of 2 hours or until the end of surgery if completed <2 hours (measurement time point 3). Furthermore, measurements were taken during any period of hypotension or hypertension with clinically indicated pharmacological intervention. Hypotension was defined as MAP below 70 mm Hg and hypertension as MAP higher than 105 mm Hg.^26^, ^27^ These recordings were treated as a single measurement and analyzed separately. Demographic data including gender, age, height and weight, ASA classification, comorbidities, and type of surgery were collected from all study participants.

### 2.4 Statistical analysis

The recommended Association for the Advancement of Medical Instrumentation (AAMI) criterion of a mean difference ≤5 mm Hg and associated SD of ≤8 mm Hg^28^ between the Nexfin‐ and invasively derived arterial pressure values was defined as the primary endpoint for the cohort of elderly perioperative patients. With 98% power at an alpha level of *α* = 5%, the minimum required sample size for the effect size of 5/8 = 0.625 was calculated to be at least 44 (two‐sided, one sample *t* test, G Power software, Düsseldorf, Germany). AAMI recommends a minimum sample size of 85 patients, although comparisons of continuous, finger‐cuff arterial pressure measurements with an invasive reference method are excluded by this standard.^28^ Thus, we deemed a sample size of 60 participants to be sufficient according to previous study protocols.^29^ Normal distribution of the outcomes was checked and verified by visual inspection of the histogram analysis. Pearson correlation analysis of measurement pairs for SAP, DAP, and MAP between the two monitoring devices was performed. Bland‐Altman analysis was used for the comparison of the paired arterial pressure measurements with calculation of the mean difference (bias) and limits of agreement (LOA) defined as the SD of the bias times 1.96.^30^ Differences were compared with the aforementioned AAMI criterion for interchangeability.^28^ The percentage error (PE) was calculated (1.96_SD_ of bias/[invasive arterial pressure/2] to quantify the relative differences between both measurement techniques as an additional statistical estimate with acceptable cut‐off values at 14.7% for SAP, 17.5% for DAP, and 18.7% for MAP as reported by Ilies et al.^15^ Finally, an analysis of concordance was conducted in order to evaluate the trending abilitity of the Nexfin system. The concordance was calculated as the percentage of measurement pairs with the same direction of change after exclusion of pairs with a change <5% in order to increase the signal‐to‐noise ratio. Based on the data points outside the exclusion zone, we calculated the concordance rate as the proportion (percentage) of concordant data pairs to all data pairs with an acceptable ability to show hemodynamic trends when the level of concordance was >92%.^31^ A *P* value of <.05 was considered as statistically significant. Statistical analysis was performed using SPSS Statistics 21 for Windows (IBM; Armonk, New York).

## 3 RESULTS

**FIGURE 2:**
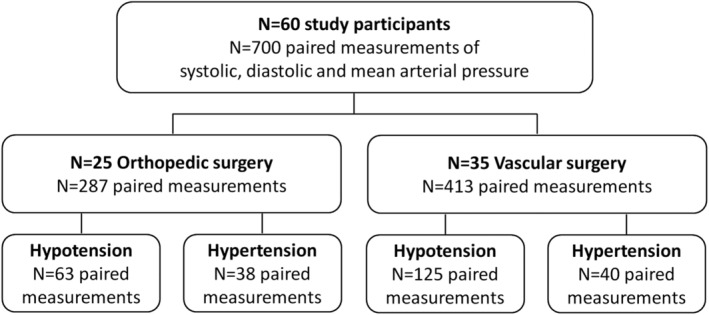
Study participant flow diagram

Sixty patients were included in the study with 25 participants undergoing orthopedic and 35 participants vascular surgery. Table 1 summarizes all relevant participant baseline characteristics and Figure 2 shows the participant flow diagram. Overall mean (SD) age was 73 (9) years, 19 participants were classified as ASA II and 41 as ASA III. The majority of the procedures in orthopedic surgery were hip replacements. Most of the vascular surgical procedures were femoral endarterectomies. Seven hundred data pairs of corresponding measurements from all 60 participants were recorded with 287 data pairs analyzed in the orthopedic surgery and 413 data pairs in the vascular surgery group, respectively. One hundred eighty‐eight measurements were performed during hypotension and 78 measurements during hypertension.

**TABLE 1:** Patient baseline characteristics

	All patients (N = 60)	Orthopedic surgery (N = 25)	Vascular surgery (N = 35)
Gender, N (%)			
Male	31 (52)	7 (28)	24 (69)
Female	29 (48)	18 (72)	11 (31)
Age (year), mean (SD)	73 (9)	77 (5)	70 (10)
Height (cm), mean (SD)	172 (9)	169 (9)	175 (9)
Weight (kg), mean (SD)	77 (16)	72 (14)	81 (17)
BMI (kg/m^2^), mean (SD)	26 (5)	25 (4)	27 (5)
ASA classification, N (%)			
II	19 (32)	13 (52)	6 (17)
III	41 (68)	12 (48)	29 (83)
Comorbidities, N (%)			
Arterial hypertension	35 (58)	13 (22)	22 (37)
Peripheral artery disease Fontaine stage ≥ II	23 (38)	1 (2)	22 (37)
Coronary artery disease	15 (25)	5 (8)	10 (17)
Diabetes mellitus	13 (22)	5 (8)	8 (13)
Atrial fibrillation	6 (10)	3 (5)	3 (5)
Type of surgery, N (%)			
Hip replacement		19 (32)	
Knee replacement		2 (3)	
Pelvic stabilization		2 (3)	
Femur shaft osteosynthesis		1 (2)	
Lower leg osteosynthesis		1 (2)	
Femoral endarterectomy			18 (30)
Carotid endarterectomy			5 (8)
Aortic aneurysm, open surgery			4 (7)
Endovascular aneurysm repair			4 (7)
Femoral popliteal bypass			2 (3)
Femorofemoral bypass			1 (2)
Femoral artery pseudoaneurysm			1 (2)

*Note*: Data are expressed as the mean ± SD or absolute and relative (in percent) frequencies, respectively.

Abbreviations: ASA, American Society of Anesthesiologists; BMI, body mass index; N, number; SD, Standard deviation.

### 3.1 Absolute and trending arterial pressure values in the total cohort

**FIGURE 3:**
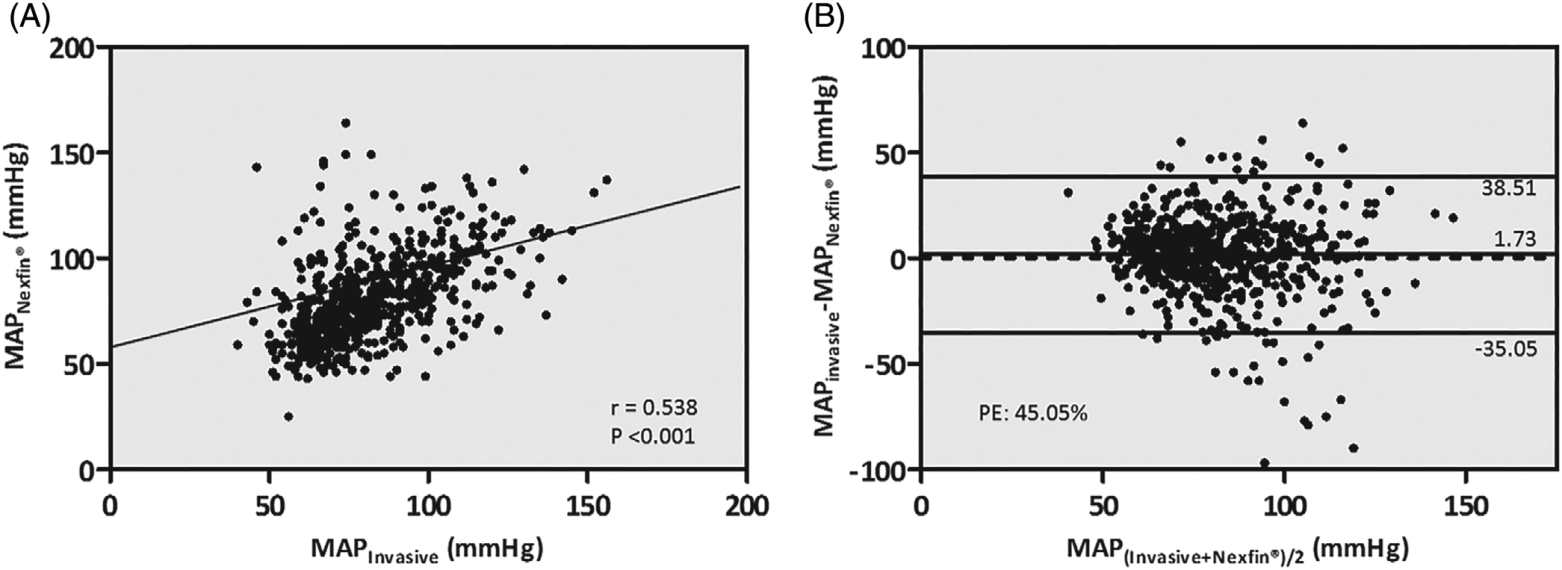
Correlation and Bland‐Altman analysis of the mean arterial pressure between Nexfin and invasive reference in the total cohort. Pearson correlation analysis (A) with coefficient and *P*‐value provided in the diagram and solid line indicating regression. (B) Shows Bland‐Altman plot of the differences vs the means of paired mean arterial pressure measurements between the invasive and noninvasive method in mm Hg. The lines correspond to the mean difference (bias) and 95% limits of agreement (SD of the bias times 1.96). In addition, the value for the calculated percentage error (PE) is displayed in the diagram

Table 2 summarizes the correlation and Bland‐Altman analyses for the absolute arterial pressure values in the total cohort. Figure 3 shows the correlation and Bland‐Altman plot exemplary for MAP in the total cohort. There was a significant positive correlation between the Nexfin‐ and arterial line‐derived pressure measurements: SAP: *r* = .542, *P* < .001; DAP: *r* = .407, *P* < .001; MAP: *r* = .538, *P* < .001. According to the Bland‐Altman analysis, Nexfin underestimated SAP and MAP with a bias (SD) of 14.43 (28.24) mm Hg and 1.73 (18.76) mm Hg, respectively and overestimated DAP with a bias of −2.40 (16.49) mm Hg. Thus, the AAMI criterion for interchangeability was not met. For SAP, PE was 43.79%, for DAP 53.78% and for MAP 45.05% exceeding the predefined cut‐off values.

**TABLE 2:** Bland‐Altman and correlation analyses in the total cohort, orthopedic and vascular surgery group and during hypertension and hypotension

	Bias (SD) (mm Hg)	Lower LOA (mm Hg)	Upper LOA (mm Hg)	PE (%)	Correlation^a^	*P* value
Systolic blood pressure (SAP)						
All data	14.43 (28.24)	−40.93	69.78	43.79	0.542	<.001
Orthopedic surgery	10.69 (30.03)	−48.18	69.56	46.14	0.517	<.001
Vascular surgery	17.08 (26.62)	−35.09	69.26	41.54	0.561	<.001
MAP >105 mm Hg	31.46 (26.44)	−20.36	83.28	29.76	0.466	<.001
MAP <70 mm Hg	7.86 (29.02)	−49.02	64.74	55.57	0.117	.109
Diastolic blood pressure (DAP)						
All data	−2.40 (16.49)	−34.73	29.92	53.78	0.407	<.001
Orthopedic surgery	−2.93 (16.32)	−34.91	29.06	51.56	0.387	<.001
Vascular surgery	−2.03 (16.63)	−34.62	30.56	55.45	0.403	<.001
MAP >105 mm Hg	7.68 (17.93)	−27.47	42.83	41.49	0.123	.289
MAP <70 mm Hg	−9.44 (17.83)	−44.39	25.51	76.08	0.004	.960
Mean arterial pressure (MAP)						
All data	1.73 (18.76)	−35.05	38.51	45.05	0.538	<.001
Orthopedic surgery	0.71 (19.47)	−37.46	38.88	45.21	0.520	<.001
Vascular surgery	2.46 (18.24)	−33.29	38.20	44.86	0.533	<.001
MAP >105 mm Hg	17.16 (19.11)	−20.30	54.61	31.75	0.277	.016
MAP <70 mm Hg	−6.40 (19.22)	−44.08	31.27	60.81	0.046	.528

Abbreviations: LOA, limits of agreement; PE, percentage error; SD, standard deviation.

a Pearson's *r*.

**FIGURE 4:**
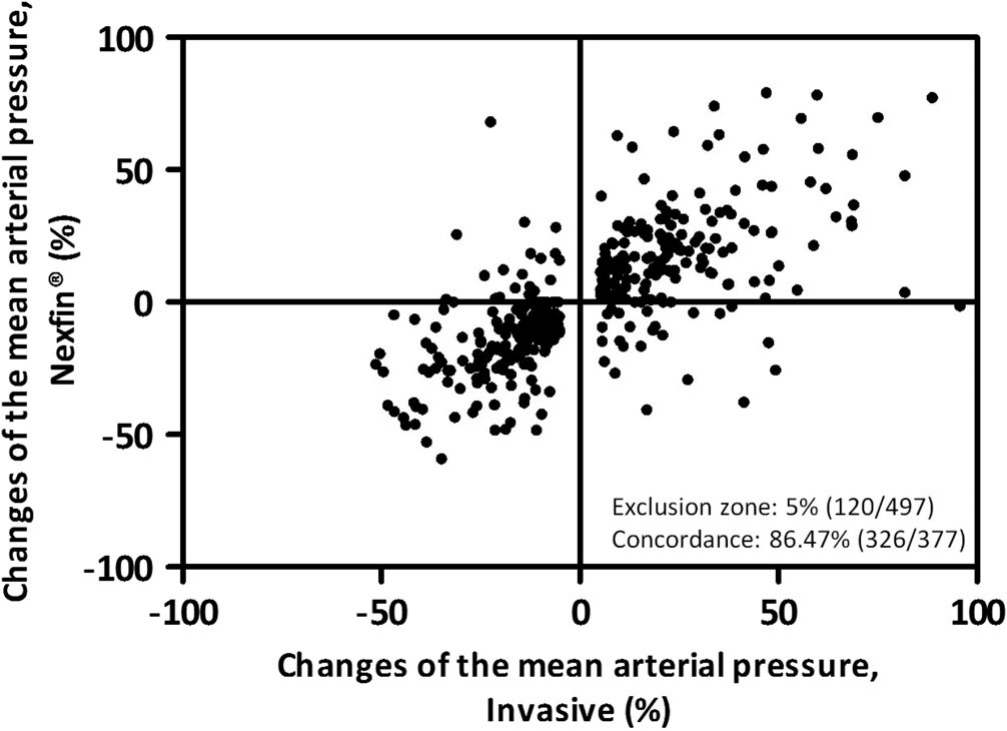
Four square plot of the concordance in mean arterial pressure changes between Nexfin and invasive reference in the total cohort. Hemodynamic trending interchangeability using a four‐quadrant plot representation of the mean arterial pressure changes in the total cohort. The number of mean arterial pressure values excluded (<5%) and the concordance rate are given in the diagram. An acceptable trending ability was assumed at a level of concordance >92%

Table 3 depicts the concordance rates of all arterial pressure value changes in the total cohort and Figure 4 the four square plot of the concordance in mean arterial pressure changes between Nexfin and invasive reference in the total cohort. The Nexfin system missed the criterion for hemodynamic trending ability in comparison to the reference system with concordance rates of 84.01% for SAP, 77.87% for DAP, and 86.47% for MAP.

**TABLE 3:** Concordance rates in the total cohort and subgroups orthopedic and vascular surgery

	All measurements	Measurements excluded^a^	Measurements included	Concordant	Non‐concordant	Concordance (%)
Systolic arterial pressure (SAP)						
All Data	499	105	394	331	63	84.01
Orthopedic surgery	205	51	154	133	21	86.36
Vascular surgery	294	54	240	198	42	82.50
Diastolic arterial pressure (DAP)						
All Data	499	133	366	285	81	77.87
Orthopedic surgery	205	51	154	129	25	83.77
Vascular surgery	294	82	212	156	56	73.58
Mean arterial pressure (MAP)						
All Data	497	120	377	326	51	86.47
Orthopedic surgery	203	49	154	138	16	89.61
Vascular surgery	294	71	223	188	35	84.30

a Changes in blood pressure <5% were excluded from concordance analysis.

### 3.2 Arterial pressure values in the orthopedic and vascular surgery groups and during hypotension and hypertension

Table 2 also presents correlation and Bland‐Altman analyses results of absolute arterial pressure values in the orthopedic and vascular surgery groups and during hypotension and hypertension. Table 3 also shows the concordance rates of trending values in the orthopedic and vascular surgery groups. Significant correlation was found for all arterial pressure measurements. Nexfin consistently underestimated SAP and MAP while DAP was slightly overestimated in the two surgery groups with no marked intergoup differences. Hemodynamic trending was observed to be best for MAP in the orthopedic surgery group, still not reaching the predefined level >92%.

**FIGURE 5:**
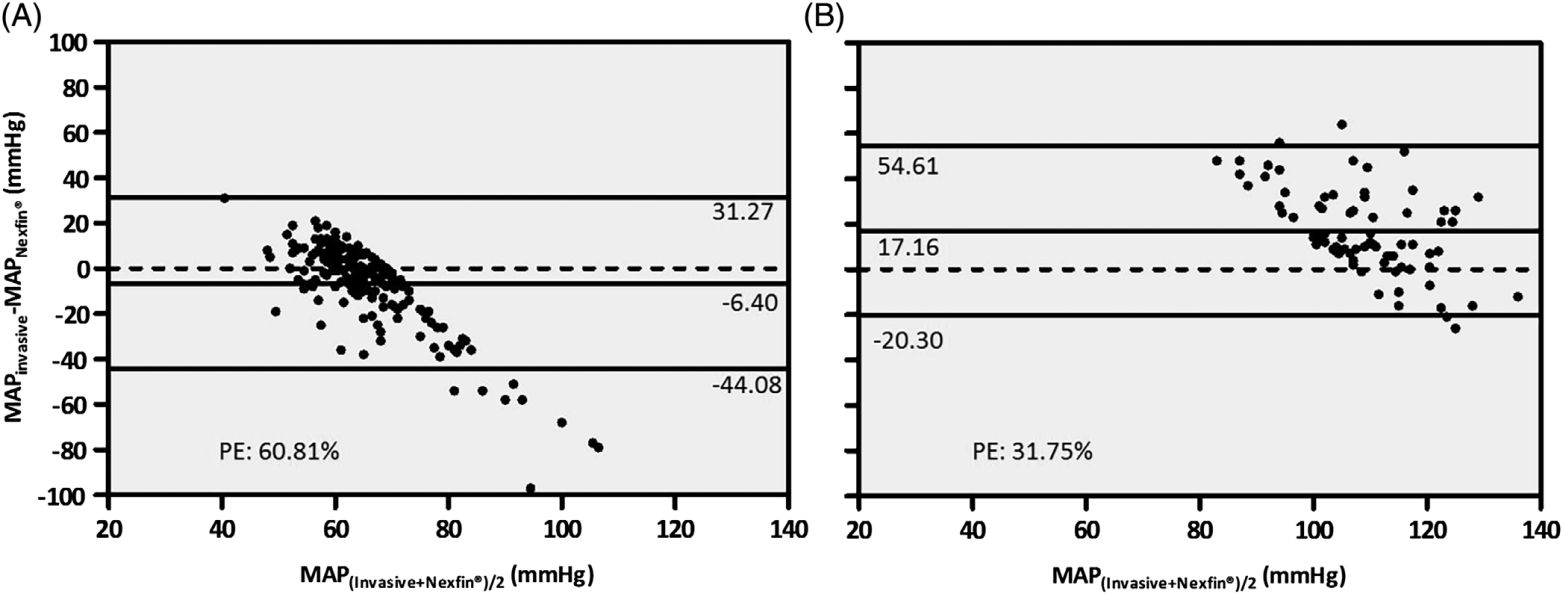
Bland‐Altman analysis of the mean arterial pressure between Nexfin and invasive reference during hypotension and hypertension. Bland‐Altman plot of the differences vs the means of paired mean arterial pressure measurements between the invasive and noninvasive method in patients with hypotension (A) and hypertension (B). The lines correspond to the mean difference (bias) and 95% limits of agreement (SD of the bias times 1.96). In addition, the value for the calculated percentage error (PE) is displayed in the diagram

During hypertension, SAP and MAP correlated significantly, whereas no significant correlation could be found for DAP between the Nexfin‐ and invasive reference system. Nexfin consistently overestimated all arterial pressure values during hypertension. Percentage error was found to be lowest during hypertension as opposed to the PE of the whole data sample (SAP: 29.76% vs 43.79%; DAP: 41.49% vs 53.78%; MAP: 31.75% vs 45.05%) but still higher than the predefined cut‐off values. During hypotension, no significant correlation could be shown for any of the three arterial pressure parameters. The bias was 7.86 mm Hg for SAP, −9.44 mm Hg for DAP, and −6.40 mm Hg for MAP. PE was highest during hypotension for all arterial pressure parameters in comparison to the total cohort: 55.57% vs 43.79% for SAP, 76.08% vs 53.78% for DAP, and 60.81% vs 45.05% for MAP. Figure 5 presents the Bland‐Altman analysis of MAP between Nexfin and invasive reference for the periods of hypotension and hypertension.

## 4 DISCUSSION

In this prospective observational cohort study arterial pressure measurement by the vascular unloading technology (Nexfin system) was compared to the invasive radial arterial line reference method in 60 elderly patients undergoing orthopedic and vascular surgery. We found that the accuracy of the vascular unloading technology during the preoperative and intraoperative phase was not sufficient. The lowest PE of all blood pressure recordings was calculated for SAP during periods of arterial hypertension while the PE was highest for DAP during arterial hypotension, still not reaching predefined cut‐off values. Furthermore, the vascular unloading method did not show an acceptable blood pressure trending capability in our perioperative setting.

A number of studies exist comparing the Nexfin technology with invasive arterial pressure measurements in different clinical settings and patient categories, including cardiac,^11^, ^32^ bariatric,^17^ and abdominal surgery^14^ as well as pediatric^33^, ^34^ and critically ill patients.^18^, ^35^, ^36^ In the latter patient group particularly, studies revealed inconsistent results where reliability of the continuous noninvasive finger blood pressure monitoring was likely hampered due to severe hypotension, required use of vasopressive agents or hypothermia. Among those studies, de Wilde et al^14^ included 19 relatively higher aged patients (mean 60.4 years) undergoing abdominal surgery and Martina et al^11^ included 50 cardiac surgery patients with a mean age of 63 years and showed that data pair values of both methods fell within the AAMI criteria.^28^

However, systematic evidence is scarce regarding whether the noninvasive vascular‐unloading method provides valid continuous arterial pressure measurements in elderly perioperative patients who exhibit a higher risk of age‐related or comorbid‐dependent vascular disorders. A recent meta‐analysis including 28 studies with 919 patients revealed that the pooled bias (SD) of continuous noninvasive compared with invasive arterial pressure measurements was −1.6 (12.2) mm Hg for SAP, 5.3 (8.3) mm Hg for DAP, and 3.2 (8.4) mm Hg for MAP which would not satisfy the standards of the AAMI guidelines.^29^ The three studies included in this meta‐analysis using the Nexfin system had an overall random‐effects pooled bias of −1.6 (8.4) mm Hg for SAP, 5.1 (6.6) mm Hg for DAP, and 3.5 (6.8) mm Hg for MAP.^11^, ^18^, ^32^ The median age was around 62 years in all 28 studies with only one study in patients with a median age 82 years. In this latter study by Schramm et al^37^ noninvasive arterial pressures measured by CNAP were compared with standard invasive arterial pressures in 29 patients undergoing elective transfemoral aortic valve implantation under analgesic sedation. CNAP accuracy was −8.3, 6.4, and 0.6 mm Hg during normotension, −20.5, 4.4, and −5.5 mm Hg during hypertension, and −4.8, 9.4, and 4.5 mm Hg during hypotension. Interestingly, the best agreement was detected in hypotensive periods as opposed to our findings with the highest PE found for DAP during hypotension. Previous studies also described a rather poor performance of the Nexfin device and pulse contour analysis techniques during hypotension.^18^, ^19^, ^38^ In a prospective study by Alfano et al,^13^ the vascular unloading technique did not correspond to oscillometric blood pressure recordings in 40 hemodynamically stable patients requiring hemodialysis. The authors suggested that the reason for the poor results of the Nexfin system might be related to the high prevalence of vasculopathy in their patient cohort with a mean age of 68.9 years.^13^ In accordance to our study, best results were shown for MAP and SAP while performance for DAP again was worst. The diastole marks the lowest part of the arterial curve between two heartbeats. So it appears likely that the precision of the diastole recordings might be even more impaired by coexisting atherosclerotic alterations in the small peripheral vessels of the fingers, compared to the radial artery of the arm. Gizdulich et al evaluated the performance of the Finapres method which is also based on the vascular unloading technique. In 53 healthy participants, they were able to show that SAP in the finger measured by the Finapres was significantly higher than in the brachial artery while DAP was concomitantly underestimated by the device.^39^ The authors interpreted their finding with a pulse wave reflection and pressure gradient due to the blood flow raising toward the vessels of the periphery. The resulting postulated model to convert finger pressure waveforms to brachial pulsation was then taken as the basis for the Nexfin algorithm.^39^ In elderly subjects and patients with signs of arteriosclerotic vascular disease, finger arterial pulse pressure is considered to be lower than the pulse pressure measured in the brachial artery, resulting in damped finger pulse pressure. In an early study in 39 patients undergoing diagnostic cardiac catheterization, O'Rourke et al showed that the mentioned pulse wave reflections lead to a rising systolic pressure in the periphery.^20^ While a diastolic wave toward the periphery was found particularly in younger participants, waveform reflection could barely be shown in older patients with atherosclerotic lesions, so that no diastolic wave was found. Thus, the accuracy of the noninvasive finger‐cuff technology might not only be hampered in patients with pre‐existing peripheral artery disease but also in the elderly due to an age‐associated arteriosclerosis with consecutive arterial stiffness and decline in vascular function, loss of arterial wall compliance and peripheral perfusion.^4^, ^5^ Van Ittersum et al already described differences between sphygmomanometric and oscillometric arterial pressure measurement devices dependent on the presence of diabetes.^40^ Van Popele et al described arterial stiffness as a cause for disagreement between an oscillometric arterial pressure monitor and a sphygmomanometer in 1808 healthy elderly subjects with the same mean age of 73 years as in our study sample.^41^ Accordingly, the Nexfin system showed a better performance during intraoperative hypertension with an overall lowest PE for all arterial pressure values in our study population, still not reaching the criterion of interchangeability.

Our study has some limitations to be addressed. Our study sample size of 60 participants deviates from the current recommendation of the AAMI, the European Society of Hypertension (ESH), and the International Organization for Standardization (ISO) collaboration that at least 85 participants are required for studies validating non‐invasive sphygmomanometers.^42^ However, there are still no specific recommendations available on how to evaluate continuous noninvasive blood pressure monitoring techniques, including methodological criteria of an adequate effect and sample size. This “gap in validation protocols” is also acknowledged in the recent AAMI/ESH/ISO collaboration statement where the task group consented that separate validation protocols need to be developed for specific functions of certain blood pressure measurement devices including continuous techniques.^42^ Another limitation is that the ipsilateral measurement of intra‐arterial pressure from the reference radial line likely introduced bias for the finger cuff photopletysmography‐based measurements of the small finger arteries.^43^ Contralateral measurements could have also introduced bias in our patient cohort due to potential differences in vessel architecture and the degree of atherosclerosis. We did not use doppler ultrasound sonography in order to evaluate peripheral arm perfusion at baseline but decided to use the same arm for both devices in accordance to previous, comparable study protocols.^11^, ^15^, ^44^ Moreover, the subanalyses of hypo and hypertension are limited due to the small sample of measurements, in particular for the analysis of hemodynamic pressure trending ability where the detected changes of the blood pressure often were to small (<5%) between two measurements for a valid calculation and subsequent exclusion as per definition. In comparison, our study is—to our knowledge—the first systematic investigation of noninvasive and invasive arterial pressure measurements in elderly patients with and without preexisting peripheral artery disease.

## 5 CONCLUSION

In conclusion, noninvasive arterial pressure measurement using the vascular unloading technique (Nexfin finger‐cuff technology) in the perioperative phase of elderly or vascular comorbid patients, respectively was not interchangeable with the gold standard of invasive arterial line measurement. Our findings underline further demand of larger clinical trials to better evaluate the useability of noninvasive measurement devices in this growing proportion of perioperative patients.

## FUNDING

No external funding was received for this study.

## CONFLICT OF INTEREST

All authors have provided information on potential conflicts of interests directly or indirectly related to the work submitted and filled out the ICMJE disclosure forms. All authors declare that they have no conflict of interest.

## AUTHOR CONTRIBUTIONS

Conceptualization: Gunnar Elke, Jochen Renner

Data Curation: Phil Klose, Ulf Lorenzen, Gunnar Elke, Jochen Renner

Formal Analysis: Phil Klose, Ulf Lorenzen, Christoph Borzikowsky, Matthias Gruenewald, Gunnar Elke, Jochen Renner

Funding Acquisition: Not applicable

Investigation: Phil Klose, Ulf Lorenzen, Rouven Berndt, Moritz Hill, Matthias Gruenewald, Gunnar Elke, Jochen Renner

Methodology: Phil Klose, Christoph Borzikowsky, Matthias Gruenewald, Gunnar Elke, Jochen Renner

Project Administration: Phil Klose, Gunnar Elke, Jochen Renner

Resources: Phil Klose, Ulf Lorenzen, Rouven Berndt, Christoph Borzikowsky, Moritz Hill, Matthias Gruenewald, Gunnar Elke, Jochen Renner

Software: Not applicable

Supervision: Gunnar Elke, Jochen Renner

Validation: Phil Klose, Ulf Lorenzen, Christoph Borzikowsky, Matthias Gruenewald, Gunnar Elke, Jochen Renner

Visualization: Phil Klose, Ulf Lorenzen, Gunnar Elke, Jochen Renner

Writing ‐ Original Draft Preparation: Phil Klose, Ulf Lorenzen, Gunnar Elke, Jochen Renner

Writing ‐ Review and Editing: Phil Klose, Ulf Lorenzen, Rouven Berndt, Christoph Borzikowsky, Moritz Hill, Matthias Gruenewald, Gunnar Elke, Jochen Renner

 All authors have read and approved the final version of the manuscript.

 The corresponding author Gunnar Elke confirms that he had full access to all of the data in the study and takes complete responsibility for the integrity of the data and the accuracy of the data analysis.

## TRANSPARENCY STATEMENT

The corresponding author Gunnar Elke confirms that the manuscript is an honest, accurate, and transparent account of the study being reported; that no important aspects of the study have been omitted; and that any discrepancies from the study as planned (and, if relevant, registered) have been explained.

## Data Availability

The data that support the findings of this study are available from the corresponding author upon reasonable request.
